# Sensitivity and specificity of double-blinded penicillin skin testing in relation to oral provocation with amoxicillin in children

**DOI:** 10.1186/s13223-020-00449-7

**Published:** 2020-07-01

**Authors:** Roxane Labrosse, Louis Paradis, Kathryn Samaan, Jonathan Lacombe-Barrios, Jean Paradis, Philippe Bégin, Anne Des Roches

**Affiliations:** 1 Pediatric Allergy and Clinical Immunology, CHU Sainte-Justine, Montreal, Canada; 2 Allergy and Clinical Immunology, Centre Hospitalier de l’Université de Montréal (CHUM), 3175 Chemin de la Côte-Sainte-Catherine, Montreal, QC H3T 1C5 Canada

**Keywords:** Penicillin allergy, Amoxicillin, Sensitivity, Specificity, Diagnostic accuracy, Skin testing

## Abstract

Current recommendations for the management of penicillin allergy are to perform penicillin skin testing (PST) with penicilloyl-polylysine (PPL) and benzylpenicillin (BP) prior to drug challenge with amoxicillin. However, the role of PST is increasingly questioned in the pediatric setting. To resolve the question of PST’s diagnostic accuracy, consecutive children with a history of non-life-threatening penicillin allergy referred to a tertiary-care allergy center were recruited to undergo double-blinded PST with PPL and BP prior to drug provocation to amoxicillin. Five of 158 participants (3.2%) presented with an immediate or accelerated reaction upon amoxicillin challenge, none of which were severe. Only one of these had positive PST (20%), compared to 15 of 153 amoxicillin tolerant participants (9.8%). The sensitivity and specificity of PST with PPL and BP for reacting upon amoxicillin challenge were 20% (95% CI: 0.5–71.6%) and 90% (95% CI: 84.4–94.4%), respectively. These results argue against the routine use of PST as a preliminary step to drug provocation with amoxicillin in this population, as it is unlikely to significantly alter pre-test probability of reacting to challenge.

## To the editor

The systematic recourse to penicillin skin tests (PST) to investigate penicillin allergy is increasingly questioned, especially in children with a history of isolated cutaneous reaction. This change was driven by both real-life cohort studies demonstrating the safety of direct challenge approaches as well as by diagnostic accuracy studies in which even patients with positive PST would undergo a confirmatory drug provocation test (DPT) [1–9]. The latter are of particular interest as they allow the calculation of sensitivity and specificity estimates, which are essential to perform cost-effectiveness analyses and extrapolate results to other clinical contexts with different pre-test probabilities. However, in all studies to date, the reference test (DPT) was universally performed with knowledge of the index test results (PST) creating a risk of interpretation bias. In addition, three of the eight studies published since 2011 did not challenge all patients with positive PST, potentially leading to verification bias.

Here, we present the results of a diagnostic accuracy study on penicillin skin testing using a prospective double-blinded design circumventing the problem of verification and interpretation bias in order. Study population consisted in patients aged 0 to 18 years reporting a history of non-life-threatening reaction to penicillin including urticaria and/or a maculopapular rash and referred for evaluation at a tertiary-care pediatric allergy center. Specific exclusion criteria included a diagnosis or a history suggestive of a severe non-IgE mediated drug allergy including severe cutaneous adverse reactions (SCARs), an active infection at time of evaluation, uncontrolled asthma, and any concurrent medication intake that could interfere with skin testing (anti-histamines, omalizumab) or place the patient at risk during challenge (beta-blockers). Patients with a history of anaphylaxis were not excluded from the outset, unless the reaction was compatible with anaphylactic shock (hypotension, altered consciousness or cardiorespiratory arrest). Consecutive patients were prospectively invited to the study starting with the 1501st patient on the waiting list to avoid regular allergy clinic appointments competing with recruitment. All patients signed informed consent forms prior to their enrollment and the study received approval from the institution’s ethics committee (CHUSJ-2013-495-3635).

Eligible participants underwent double-blinded skin testing using a method described previously [10]. Blinded PST reagents were prepared by an unblinded nurse (professional 1) in a random order that was sealed in an envelope, to be opened only at time of analysis. Testing was performed by a second nurse (professional 2) who was blinded to reagent order. Intradermal testing was performed on the volar face of the arm with penicilloyl-polylysine (PPL) 6.0 × 10^−5^ M (PRE-PEN^®^, AllerQuest, LLC, Plainville, CT), benzylpenicillin (BP) 10,000 UI/mL (Fresenius Kabi Canada Ltd, Richmond Hill, Ontario), BP 1000 IU/mL and saline, in random order. A prick test with histamine was used as positive control. Skin tests were read after 15 min by an allergist also blinded to the order of the tests (professional 3). The tests were then covered with towels to avoid influencing the second blinded allergist supervising the drug provocation (professional 4).

The graded drug provocation to amoxicillin (45/mg/kg) was performed in three incremental steps: 1/100, 1/10, and full dose at 30 min intervals followed by 1 h of observation to identify signs of IgE-mediated reaction. Participants were called to ensure the absence of accelerated reaction in the following 48 h. The study planned for an initial sample size of 300 patients, based on the assumption of positive predictive value (PPV) of 25% and rate of positive PST of 10% [5]. PST was considered positive if either the PPL or BP 10,000 UI/mL generated a mean wheal diameter that was 3 mm greater than the negative control, with flare, as per practice parameter [11]. The sensitivity and specificity of PST were calculated in light of DPT results, which constitutes the gold standard for diagnosis of penicillin allergy. There were no pre-specified plans for subgroup analysis or rules for early termination.

**Fig. 1:**
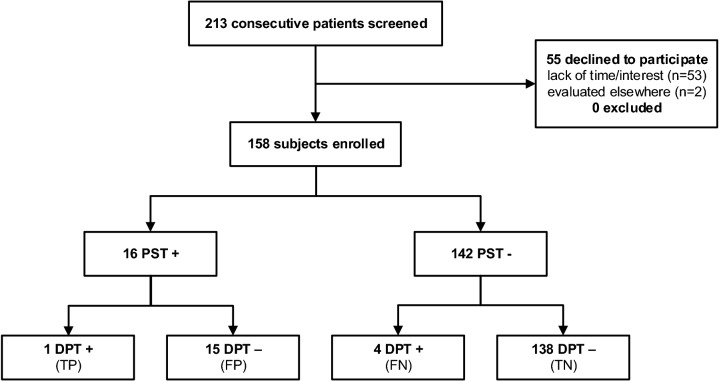
Flowchart of patients enrolled in study with penicillin skin testing (PST) and drug provocation test (DPT) results

Between October 2013 and June 2015, 213 patients were approached to participate in the study, of which 158 were enrolled and none of whom met exclusion criteria (Fig. 1). Demographic and clinical characteristics of participants are presented in Table 1. All participants completed the study.

**Table 1:** Participants’ characteristics

Number of patients	n = 158
Age (years)	
Mean (range)	5.5 (0.7–18.1)
Gender, n (%)	
Male	82 (51.9)
Personal history of atopy, n (%)	
Any atopic condition	71 (44.9)
Atopic dermatitis	47 (29.7)
Allergic rhinitis	29 (18.4)
Asthma	32 (20.3)
Food allergy	8 (5.1)
Personal history of other drug allergy, n (%)	45 (28.5)
Family history of drug allergy, n (%)	50 (31.6)
Molecule of initial reaction, n (%)	
Amoxicillin	137 (86.7)
Amoxicillin/clavulinic acid	13 (8.2)
Presumed amoxicillin	6 (3.8)
Penicillin V	1 (0.6)
Ampicillin	1 (0.6)
Indication for antibiotic	
Otitis media	80 (50.6%)
Pharyngitis	15 (9.5%)
Pneumonia/bronchitis	10 (6.3%)
Sinusitis	3 (1.9%)
Scarlet fever	3 (1.9%)
Other	11 (7.0%)
Unknown	36 (22.8%)
Allergic reaction, n (%)	
Rash	158 (100.0)
Urticaria	59 (37.3)
Maculopapular rash	89 (56.3)
Macular rash	7 (4.4)
Undefined rash	2 (1.3)
Angioedema	11 (7.0)
Cough/bronchospasm	2 (1.3)
Vomiting	5 (3.2)
Anaphylaxis	0 (0.0)
Timing of allergic reaction, n (%)	
Within < 24 h	27 (17.1)
After > 24 h	99 (62.7)
Unknown	32 (20.3)
Time elapsed between initial reactions and allergy workup, years (median, interquartile range)	1.6 (0.7–4.8)

The study was terminated following this interim analysis for cause of futility. Of the 16 patients with a positive intradermal PST (8.2%), only one presented an immediate (< 6 h) reaction on drug provocation (Table 2). In those with negative PST, two presented an immediate and two presented an accelerated (6 h-48 h) reaction upon drug provocation. All reactions were mild and limited to the skin and responded promptly to anti-histamine treatment. Delayed PST reading was negative in all patients. The predictive value of PST was 6.3% (95% CI: 0.4–26.3%). The observed sensitivity and specificity for IgE-mediated reactions (immediate or accelerated) were 20% (95% CI 0.5–71.6%) and 90% (95% CI 84.4–94.4%), respectively. The decision to terminate the study was made by the investigators following discussion with the ethics committee and was based on the rationale that even if sensitivity had been at the upper limit of the 95% CI (i.e. 70%), with the observed prevalence of true allergy (3.2%) PST would still need to be performed in 39 patients in order to prevent a single mild reaction on challenge.

**Table 2:** Participants with positive intra-dermal skin tests

Sex	Age (years)	Delay since reaction (years)	Culprit drug	BP (mm)		PPL (mm)	NS (mm)	DPT
				IU/mL				
				10^4^	10^3^			
True positive								
F	8	0.7	AXO	5	–	–	–	Urticaria and angioedema < 1 h post-DPT
False positives								
M	5	1.3	AXO	8	–	–	–	–
M	2	0.6	AXO	7	–	–	–	–
F	5	1.0	AXO	6	–	–	–	–
F	10	9.2	AXO	5	–	–	–	–
M	11	5.7	AXO	4	–	–	–	–
M	2	0.5	AXO	7	–	–	–	–
M	2	0.7	AXO	4	–	–	–	–
M	3	1.8	AXO	7	–	–	–	–
M	2	0.5	AXO	8	4	–	–	–
M	11	6.3	AXO	6	9	–	–	–
F	18	17.6	AXO	3	3	5	–	–
F	13	11.6	AXO	10	–	8	5	–
F	2	1.0	CLAV	–	3	4	–	–
M	17	11.3	AXO	–	–	5	–	–
M	2	0.2	AXO	–	–	7	–	–
False negatives								
F	2	0.4	AXO	–	–	–	–	Urticaria < 1 h post-DPT
M	2	0.5	AXO	–	–	–	–	Urticaria < 1 h post-DPT
F	3	1.8	AXO	–	–	–	–	Urticaria at 24 h post-DPT
F	2	0.7	AXO	–	–	–	–	Urticaria at 12 h post-DPT

*BP* benzylpenicillin, *PPL* penicilloyl-polylysine, *NS* normal saline, *DPT* drug provocation test, *MDM*; *AXO* amoxicillin, *CLAV* amoxicillin + clavulinic acid

The main strength of the study stems from its robust methodology, which completely eliminates potential risks of verification and interpretation bias and allows for an impartial confirmation of previous estimates. The scarcity of true allergy in the pediatric population with a history of non-severe cutaneous reaction was also confirmed. The downside is that the low event rate also leads to large confidence interval around estimates. This phenomenon is further aggravated when excluding accelerated reactions, with a sensitivity estimate of 33% and a 95% confidence interval of 0.8 to 90.5%. Future studies will need to be conducted in populations with higher rates of true allergy to improve the precision of these estimates.

The study also underscores the lack of reliability of skin testing, best exemplified by a patient with a discordant positive result to low concentration of PPL and negative result to a high concentration of PPL. Because, allergy skin test sizes are known to vary with repeat testing, some studies have performed these in triplicate to improve reliability. Such an approach would not however be representative of real-life practice, where tests are not replicated. If they were, the improved diagnostic accuracy would then have to be interpreted in light of the increased cost of reagents.

One limitation of this study is the lack of intradermal testing with amoxicillin, which is not available in its injectable form in Canada. One could assume that adding amoxicillin could have increased test sensitivity, although this was not systematically the case in other studies including it [1, 5–8]. Addition of reagents the PST panel is also bound to decrease test specificity and increase cost of testing, which needs to be taken into consideration.

Although most international guidelines continue to recommend systematic PST for the assessment of penicillin allergy, recent guidelines from Canada [12] and the UK [13] argue against skin testing in children at low risk for penicillin allergy, especially in those with nonimmediate reactions which are often the result of the underlying infectious process rather than true drug hypersensitivity. This study has important implications, since it suggests that in children with nonsevere amoxicillin allergy, including proven IgE-mediated reactions, skin testing does not seem to be an effective screening tool. Consequently, these patients might benefit from undergoing a direct graded DPT, without prior skin testing.

In conclusion, using a novel double-blinded skin-testing approach, this study was able to validate the poor accuracy of PST for the diagnosis of amoxicillin allergy, while controlling for verification and interpretation bias. This further supports the futility of PST prior to amoxicillin provocation in this population.

## Data Availability

The authors confirm that the data supporting the findings of this study are available within the article.

## Abbreviations

BP: Benzylpenicillin
IgE: Immunoglobulin E
NPV: Negative predictive value
PPL: Penicilloyl-polylysine
PPV: positive predictive value
PST: Penicillin skin testing
